# A Recurrent Neural Network-Based Method for Dynamic Load Identification of Beam Structures

**DOI:** 10.3390/ma14247846

**Published:** 2021-12-18

**Authors:** Hongji Yang, Jinhui Jiang, Guoping Chen, M Shadi Mohamed, Fan Lu

**Affiliations:** 1 State Key Laboratory of Mechanics and Control of Mechanical Structures, Nanjing University of Aeronautics and Astronautics, Nanjing 210016, China; yanghongji@nuaa.edu.cn (H.Y.); gpchen@Nuaa.edu.cn (G.C.); 2 School of Energy, Geoscience, Infrastructure and Society, Heriot-Watt University, Edinburgh EH14 4AS, UK; 3 Institute of Acoustics, Chinese Academy of Sciences, Beijing 100190, China; lufan@mail.ioa.ac.cn

**Keywords:** dynamic load identification, time-domain solution, simply supported beam, recurrent neural network, long short-term memory

## Abstract

The determination of structural dynamic characteristics can be challenging, especially for complex cases. This can be a major impediment for dynamic load identification in many engineering applications. Hence, avoiding the need to find numerous solutions for structural dynamic characteristics can significantly simplify dynamic load identification. To achieve this, we rely on machine learning. The recent developments in machine learning have fundamentally changed the way we approach problems in numerous fields. Machine learning models can be more easily established to solve inverse problems compared to standard approaches. Here, we propose a novel method for dynamic load identification, exploiting deep learning. The proposed algorithm is a time-domain solution for beam structures based on the recurrent neural network theory and the long short-term memory. A deep learning model, which contains one bidirectional long short-term memory layer, one long short-term memory layer and two full connection layers, is constructed to identify the typical dynamic loads of a simply supported beam. The dynamic inverse model based on the proposed algorithm is then used to identify a sinusoidal, an impulsive and a random excitation. The accuracy, the robustness and the adaptability of the model are analyzed. Moreover, the effects of different architectures and hyperparameters on the identification results are evaluated. We show that the model can identify multi-points excitations well. Ultimately, the impact of the number and the position of the measuring points is discussed, and it is confirmed that the identification errors are not sensitive to the layout of the measuring points. All the presented results indicate the advantages of the proposed method, which can be beneficial for many applications.

## 1. Introduction

External excitation is the main source of basic dynamic data for many engineering applications, such as structural dynamic characteristics, vibration response analysis, health monitoring, vibration fatigue analysis and vibration fault diagnosis [1,2,3,4,5], among others. In the majority of these applications, measuring dynamic loads directly is not possible. Such measurements are often limited by the accuracy of the test technology and the complexity of large equipment structures where force sensors are difficult to install. How to identify various forms of dynamic load is a fundamental question that has been discussed in numerous studies. However, the traditional dynamic load identification methods are deeply dependent on determining the dynamic characteristics of a structure first [6].

Dynamic load identification is usually achieved in the time or the frequency domain. In the frequency domain, it is necessary to inverse the structural dynamic characteristics matrix, which is often ill conditioned. This can result in a major impact on the accuracy, especially in noisy environments [7]. Similarly, in the time domain, the identification results often diverge or deviate due to the errors accumulation over the time span of interest. Therefore, the accuracy of dynamic load identification is difficult to guarantee and the structural dynamic characteristic information is difficult to obtain, especially for large complex structures [8,9]. However, this remains an important research field where many industrial and academic experts are committed to promoting the study of dynamic load identification [10].

Constructing the inverse model of a vibration response and the associated external excitation is the basic premise of dynamic load identification. With decades of development since the 1970s [11], dynamic load identification has developed in three diverse directions, namely, frequency-domain identification methods, time-domain identification methods and intelligent algorithms. Among these, frequency-domain methods are the earliest, and are considered by many to be the classical methods. These methods are usually based on building an inverse model between the response and the excitation [12,13]. Frequency-domain methods mainly rely on either the direct inversion, the least square approach or the modal coordinate transformation method. All these methods involve inverting the matrix of the frequency response function, which more often than not suffers from severe ill-conditioning issues [14,15]. Although scholars have studied numerous regularization methods for ill-conditioned problems [16,17,18,19,20], there are still many difficulties with the implementation details of frequency-domain methods. Nevertheless, frequency-domain methods are still considered by industrial users to be more mature than the other methods. Hence, they are intensively used to identify excitations in many engineering applications, such as wind load, six-force-factor and the load on mining machinery [21,22,23].

Compared to the frequency domain, time-domain methods can be considered to be more intuitive as they take into account time as a variable. Utilizing the model parameters of a structure to establish the inverse model of the system and identifying the input based on the output of the system is the general procedure of time-domain methods [24]. Nowadays, the existing time-domain methods are basically based on modal decomposition technology and Duhamel integral technology [25,26,27,28,29]. Most of the time-domain methods cannot identify dynamic loads with high accuracy due to the restrictions imposed by several factors, such as ill-posedness, cumulative error and the unclear parameters of the studied dynamic system [30,31,32]. Additionally, noisy environments, complex structures, structures with repeated frequencies, as well as the resonance and anti-resonance points of a structure, can also have a great impact on the identification accuracy [33,34,35].

As early as 1998, Cao et al. [36] used neural networks to solve the dynamic load identification problem facing aircraft wings. Nevertheless, neural network had not been further developed in load identification owing to limitations in computing technologies. With the rapid development of deep learning in recent years, more and more intelligent algorithms have been developed for dynamic load identification. For instance, Liu et al. [37] presented a novel method based on support vector regression to establish the uncertain load caused by heterogeneous responses. Wang et al. [38] proposed a deep regression adaptation network method with model transfer learning to improve the accuracy and efficiency of neural networks for dynamic load identification. Zhou et al. [39] proposed a novel impact load identification method based on a deep recurrent neural network for nonlinear structures. Cooper et al. [40] developed an artificial neural network model to predict the static load applied on a wing rib. All the above-mentioned literature shows an increasing trend which suggests that intelligent algorithms will be very important for the future of dynamic load identification.

Given the features of dynamic load identification, it can be classified as a regression problem of deep learning. Both a vibration response signal and an external excitation signal are considered to involve change over time. The inverse model of a single-channel response or a multi-channel response and a force signal can be established, which is the core idea of load identification. According to different data characteristics and final objectives, different deep learning models can be applied under different engineering scenarios. For instance, multilayer perceptron (MLP) is widely used in table data processing, convolutional neural networks play an important role in image processing and support vector machines have great advantages in limited samples learning. Given that the initial vibration data are often collected in the time domain, we propose using a recurrent neural network (RNN) for dynamic load identification without needing the structural dynamic characteristics. RNN is essentially a model for establishing the nonlinear relationship between multiple variables [41], which is suitable for processing time-domain data. Additionally, in an RNN model, the input of the current time and the output of the previous time can be effectively connected by a basic operation [42]; that is, the amplitude of vibration response data at each time point can be related through time. The RNN models are suitable for solving the identification problem faced by time series models, of which dynamic load identification is a representative problem. To solve the problem of gradient explosion or gradient disappearance in an ordinary RNN model [43], we propose applying the concept of the long short-term memory (LSTM) here. Moreover, bidirectional long short-term memory (BLSTM) is also introduced, which can connect previous and future information in the time domain. These variants of the RNN model are useful for multi-series prediction problems. Compared with RNN, the structure of LSTM is more complex. Specifically, LSTM adds a structure that can remember longer sequences of information, adds an input gate, a forgetting gate and an output gate, and reduces the probability of gradient disappearance or gradient explosion [44]. In other fields, LSTM was initially developed for natural languages processing. More recently, its application in other fields has also been explored by several scholars. For instance, Graves et al. [45] used bidirectional long short-term memory (BLSTM) networks to classify the framewise phoneme. Ordóñez et al. [46] proposed a generic deep framework for activity recognition based on convolutional and LSTM-recurrent units to capture the temporal dynamics of human activity recognition. Han et al. [47] proposed a novel architecture of neural networks, referred to as the long short-term neural network (LSTM NN), to capture nonlinear dynamic traffic in an effective manner. Liu et al. [48] proposed a tree structure-based traversal method, and introduced a new gating mechanism within LSTM to learn the reliability of the sequential input data. Li et al. [49] deployed LSTM networks to predict out-of-sample directional movements for the constituent stocks of the S&P500 from 1992 until 2015.

Dynamic load identification is important to several areas of system engineering, including forward dynamics, system modelling, parameter identification and the inverse problem, among others. The error introduced in any segment will greatly affect the ultimate identification result, which is similar to other fuzzy fields [50,51,52]. Moreover, different material properties will affect the solution for the problem relating to structural dynamic characteristics [53,54,55,56], which makes identification difficult. Scholars usually use the metaphor of the “black box” to describe the problem of dynamic load identification and neural networks. Here, we combine the two black box problems to reduce the difficulty of dynamic load identification.

After considering a range of different aspects, we believe that deep learning has great potential in the field of dynamic load identification. However, there is no complete dynamic load identification theory based on deep learning. Starting with RNN and LSTM, we establish a complete dynamic load identification system in order to apply this method to engineering practice, and to successfully identify common dynamic loads. In this approach, sinusoidal, impulse and random excitations are identified on a simply supported beam. Furthermore, the effects of changing the network structure and the hyperparameters on the identification results are also evaluated. We show the possibility of using this method for multi-points excitations. To our satisfaction, we find that the identification results are not sensitive to the layout of measuring points. This is a significant advantage that can be beneficial if the proposed method is extended to other engineering applications.

## 2. Dynamic Load Identification Framework Based on RNN

### 2.1. Basic Description

**Figure 1:**
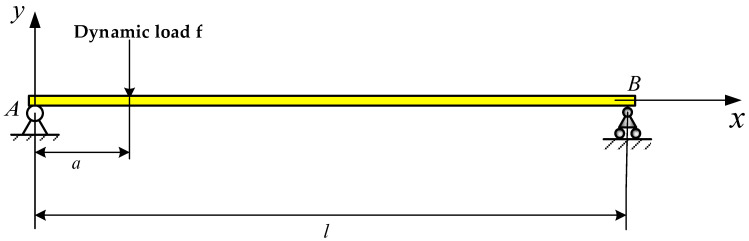
Dynamic load identification diagram of a simply supported beam.

A beam, which is the most primitive type of continuous structure, can be an efficient simplification for different applications. A Bernoulli–Euler beam with simply supported boundary conditions and a homogeneous material is shown in Figure 1. The cross-section area, the density and the elastic modulus are given as A, ρ and E, respectively. The moment of inertia of the interface is I.

The dynamic equation [25] of the beam can be written as:

(1) $$EI\frac{\partial^{4}u}{\partial x^{4}}+EIc_{0}\frac{\partial u}{\partial t}+EIc_{1}\frac{\partial^{5}u}{\partial t\partial x^{4}}+\rho A\frac{\partial^{2}u}{\partial x^{2}}=f$$

where EI is the section stiffness, ρA is the mass per unit length, u is the transverse deformation, $c_{0}$ is the viscous damping coefficient of an external medium, $c_{1}$ is the internal damping coefficient and f is the external load on the beam. It is assumed that the beam is subjected to a concentrated simple harmonic load. Then, the above differential equation [57] can be depicted in modal coordinates as:

(2) $${\overset{••}{q}}_{j}+2\xi_{j}{\bar{\omega }}_{j}^{2}{\overset{•}{q}}_{j}+{\bar{\omega }}_{j}^{2}q_{j}=Q_{j}(t)$$

in which ${\bar{\omega }}_{j}^{2}$, $\xi_{j}$ and $q_{j}$ are the natural frequency, damping ratio and modal coordinates, respectively. The terms in this equation are defined by:

(3) $$\begin{array}{l} 2\xi_{j}{\bar{\omega }}_{j}^{2}=c_{0}\frac{EI}{\rho A}+c_{1}{\bar{\omega }}_{j}^{2}\\ Q_{j}(t)=f(x_{a},t)\varphi_{j}(x_{a})\sin(\omega t)/M_{j}\\ M_{j}=\int_{0}^{L}\rho A\varphi_{j}^{2}(x)dx \end{array}$$

Here, $M_{j}$, $\varphi_{j}\left(\omega \right)$ and $\varphi_{j}\left(x_{a}\right)$ are the modal mass, modal shape and the value of the *j*th modal shape, respectively. Additionally, $f\left(x_{a}\right)$ is the load value at point *a* and $Q_{j}\left(t\right)$ is the modal force. Solving Equation (2), the convolution integral form of the solution can be detailed as:

(4) $$q_{j}(t)=\int_{0}^{t}h_{j}(t-\tau )Q_{j}(\tau )dt$$

Therefore, we can derive the expression of displacement response as:

(5) $$u(x,t)=\frac{2}{\rho Al}\sum_{n=1}^{\infty }\sin(\frac{n\pi x}{l})[f(x_{a},t)\sin(\frac{n\pi x_{a}}{l})+\int_{0}^{t}\overset{\cdot \cdot }{h_{n}}(t-\tau )f(x_{a},t)\sin(\frac{n\pi x_{a}}{l})d\tau ]$$

where $\overset{\cdot \cdot }{h_{n}}(t)=\frac{1}{\omega_{n}^{\prime }}e^{\xi_{n}\omega_{n}t}\{\left[{\left(\xi_{n}\omega_{n}\right)}^{2}-{\left(\omega_{n}^{\prime }\right)}^{2}\right]\sin(\omega_{n}^{\prime }t)+(-2\xi_{n}\omega_{n}\omega_{n}^{\prime })\cos(\omega_{n}^{\prime }t)\}$.

The load is fitted by a set of orthogonal polynomials [58,59], which can be written as:

(6) $$f(x_{a},t)=\sum_{i=1}^{\infty }a_{i}P_{i}(t)$$

where $a_{i}$ and $P_{i}\left(t\right)$ are the coefficient of orthogonal polynomials and the *i*th element of orthogonal polynomials, respectively. When the fitting accuracy can be satisfied, the last equation can be rewritten as:

(7) $$f(x_{a},t)=\sum_{i=1}^{\infty }a_{i}P_{i}(t)=\left\{\begin{array}{cccc} P_{1} & P_{2} & \cdots & P_{n_{f}} \end{array}\right\}\left\{\begin{array}{c} a_{1}\\ a_{2}\\ \vdots \\ a_{n_{f}} \end{array}\right\}$$

Assume that the number of quantities to be identified, the number of samples in the time domain and the sampling time are $n_{f}$, $N_{s}$ and $t_{s}$, respectively. The following relationships can be derived as:

(8) $$\left\{\begin{array}{c} {\overset{\cdot \cdot }{u}}_{k}^{t_{1}}\\ {\overset{\cdot \cdot }{u}}_{k}^{t_{2}}\\ \vdots \\ {\overset{\cdot \cdot }{u}}_{k}^{t_{s}} \end{array}\right\}=\frac{1}{M}\sum_{n=1}^{\infty }S_{nk}\left[\begin{array}{cccc} H_{1}^{p_{t_{1}}} & H_{2}^{p_{t_{1}}} & \cdots & H_{n_{f}}^{p_{t_{1}}}\\ H_{1}^{p_{t2}} & H_{2}^{p_{t_{2}}} & \cdots & H_{n_{f}}^{p_{t_{2}}}\\ \vdots & \vdots & \cdots & \vdots \\ H_{1}^{p_{ts}} & H_{2}^{p_{ts}} & \cdots & H_{n_{f}}^{p_{ts}} \end{array}\right]\left\{\begin{array}{c} a_{1}\\ a_{2}\\ \vdots \\ a_{n_{f}} \end{array}\right\}$$

where ${\ddot{u}}_{k}^{t_{s}}$ is the value of $\ddot{u}\left(x,t\right)$ on a point k at the time t and $S_{nk}$ is the value of $S_{n}$ at the point k. Subsequently, $H_{n_{f}}^{P_{t_{s}}}$ is the value of $H_{n_{f}}^{P}$ at the time $t_{s}$. In addition, $S_{n}=\sin\left(\frac{n\pi x}{l}\right)\sin\left(\frac{n\pi x_{a}}{l}\right)$ and $H_{n_{f}}^{P}=P_{n_{f}}+\int_{0}^{t}\ddot{h}\left(h-\tau \right)d\tau$, which is the element of transfer function. In Equation (8), the items to be identified are $a_{1},\;a_{2},\;\ldots ,\;a_{n_{f}}$. Eventually, Equation (8) can be abbreviated as:

(9) $$\left\{{\tilde{u}}_{t_{s}\times 1}\right\}=\left[{\tilde{H}}_{t_{s}\times n_{f}}\right]\left\{A_{nf\times 1}\right\}$$

When $N_{s}=n_{f}$, $\tilde{H}$, which is the transfer function, can be inversed directly and the coefficients of the equations are calculated as:

(10) $$\left\{A\right\}={\left[\tilde{H}\right]}^{-1}\left\{\tilde{u}\right\}$$

While $N_{s}>n_{f}$, the coefficients of the system of contradictory equations can be obtained through the generalized inverse solution of the least squares, which can be derived as:

(11) $$\left\{A\right\}={\left[{\left[\tilde{H}\right]}^{T}\left[\tilde{H}\right]\right]}^{-1}{\left[\tilde{H}\right]}^{T}\left\{\tilde{u}\right\}$$

Equation (11) is the mathematical model of dynamic load identification based on the generalized orthogonal polynomial under the action of time-varying concentrated force. In general, measuring the acceleration is easier than the displacement or the velocity. Therefore, in this paper, we construct the identification model of the beam structure based on the acceleration.

It can be seen from the above derivation that most time-domain identification methods need the model parameters of the structure. Obtaining an impulse response function for complex structures is often an exhausting process. Due to this difficulty and the characteristics of RNN models, this paper combines a deep RNN model with dynamic load identification to reduce the difficulty of load identification in engineering applications.

### 2.2. Recurrent Neural Network Implementation

The selection of training data is the first step that needs to be considered for deep learning models [60]. With dynamic load identification, the application of RNN requires identifying the load type in advance. The types to be considered here include a simple harmonic load, an impact load, a random load or a superposition of sinusoidal loads. These types in general cover most of the dynamic loads to be identified in engineering applications. Taking the dynamic load of a piece of rotating machinery as an example, its dynamic load is generally a quasi-harmonic signal with the motor frequency as the main frequency and the coupling frequency of other parts or noise interference as the auxiliary [61]. Therefore, when the motor parameters of a piece of rotating machinery are known, the shape of the force signal acting on the structure by the motor can be roughly inferred. Hence, the load type can be assumed, and the recorded dynamic load can be used for training. The vibration response and the assumed dynamic load are the input in this case. Repeated training is carried out to establish the inverse model. Additionally, the historical data of real dynamic loads can also be used as the input for RNN. Taking the impact excitation as an example, we can obtain multiple impact loads by continuously knocking and recording the vibration response. Subsequently, the load data of the previous times can then be used as training data to identify the dynamic impact loads of the later impacts [39].

**Figure 2:**
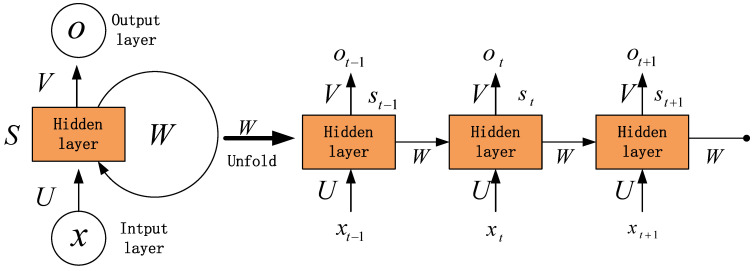
RNN structure of a single hidden layer.

The structure of a single hidden layer in RNN is shown in Figure 2, in which x, s and o are the discrete vibration response time series, the output of the hidden layers and the output, respectively. *U*, *V* and *W* are the weights of the input layer to the hidden layer, the hidden layer to the output layer and the self-recursion, respectively. Hence, the output of the hidden layer [62] can be written as:

(12) $$s_{t}=f(Ux_{t}+Ws_{t-1})$$

where *f* is the activation function. Moreover, the output of output layer can be described as:

(13) $$o_{t}=g(Vs_{t})$$

in which *g* is also an activation function.

The propagation process of a single hidden layer can be defined as:

(14) $$\begin{array}{l} o_{t}=g(Vs_{t})\\ =Vf(Ux_{t}+Ws_{t-1})\\ =Vf(Ux_{t}+Wf(Ux_{t-1}+Ws_{t-2}))\\ =Vf(Ux_{t}+Wf(Ux_{t-1}+Wf(Ux_{t-2}+Ws_{t-3})))\\ =Vf(Ux_{t}+Wf(Ux_{t-1}+Wf(Ux_{t-2}+Wf(Ux_{t-3}+\ldots )))) \end{array}$$

Therefore, the predicted dynamic load can be derived using Equation (14). Stacking the single hidden layer in Figure 2 to establish a deep network, the output can be written as:

(15) $$\begin{array}{l} o_{t}=g(V^{i}s_{t}^{i}+V^{i}s_{t}^{i})\\ s_{t}^{i}=f(U^{i}s_{t}^{i-1}+W^{i}s_{t-1})\\ s_{t}^{i-1}=f(U^{i-1}s_{t}^{i-2}+W^{i-1}s_{t-1})\\ \vdots \\ s_{t}^{1}=f(U^{1}x_{t}+W^{1}s_{t-1}) \end{array}$$

The process of forward propagation can be described using Equation (12), which in matrix form is:

(16) $$\left[\begin{array}{c} s_{1}^{t}\\ s_{2}^{t}\\ \vdots \\ s_{n}^{t} \end{array}\right]=f(\left[\begin{array}{cccc} U_{11} & U_{12} & \cdots & U_{1m}\\ U_{21} & U_{22} & \cdots & U_{2m}\\ \vdots & \vdots & \vdots & \vdots \\ U_{n1} & U_{n2} & \cdots & U_{nm} \end{array}\right]\left[\begin{array}{c} x_{1}\\ x_{2}\\ \vdots \\ x_{m} \end{array}\right]+\left[\begin{array}{cccc} W_{11} & W_{12} & \cdots & W_{1m}\\ W_{21} & W_{22} & \cdots & W_{2m}\\ \vdots & \vdots & \vdots & \vdots \\ W_{n1} & W_{n2} & \cdots & W_{nm} \end{array}\right]\left[\begin{array}{c} s_{1}^{t-1}\\ s_{2}^{t-1}\\ \vdots \\ s_{n}^{t-1} \end{array}\right])$$

The back propagation calculation of RNN has two directions, namely, back propagation along time and along the layer. Moreover, the first kind of process of forward propagation [63] can be abbreviated as:

(17) $$\begin{array}{l} net_{t}=Ux_{t}+Ws_{t-1}\\ s_{t-1}=f(net_{t-1}) \end{array}$$

in which $net_{t}$ is an alternative parameter to $s_{t}$. Specifically, the relationship between two adjacent moments of $net_{t}$ can be written as:

(18) $$\frac{\partial net_{t}}{\partial net_{t-1}}=\frac{\partial net_{t}}{\partial s_{t-1}}\frac{\partial s_{t-1}}{\partial net_{t-1}}$$

where the two terms to the right of the equal sign can be described with the following equations.

Substituting Equation (19) into Equation (18), we can obtain the following:

(19) $$\begin{array}{l} \frac{\partial net_{t}}{\partial s_{t-1}}=\left[\begin{array}{cccc} \frac{\partial net_{1}^{t}}{\partial s_{1}^{t-1}} & \frac{\partial net_{1}^{t}}{\partial s_{2}^{t-1}} & \cdots & \frac{\partial net_{1}^{t}}{\partial s_{n}^{t-1}}\\ \frac{\partial net_{2}^{t}}{\partial s_{1}^{t-1}} & \frac{\partial net_{2}^{t}}{\partial s_{2}^{t-1}} & \cdots & \frac{\partial net_{2}^{t}}{\partial s_{n}^{t-1}}\\ \vdots & \vdots & \vdots & \vdots \\ \frac{\partial net_{n}^{t}}{\partial s_{1}^{t-1}} & \frac{\partial net_{n}^{t}}{\partial s_{2}^{t-1}} & \cdots & \frac{\partial net_{n}^{t}}{\partial s_{n}^{t-1}} \end{array}\right]=\left[\begin{array}{cccc} W_{11} & W_{12} & \cdots & W_{1n}\\ W_{21} & W_{22} & \cdots & W_{2n}\\ \vdots & \vdots & \vdots & \vdots \\ W_{n1} & W_{n2} & \cdots & W_{nn} \end{array}\right]=W\\ \frac{\partial s_{t-1}}{\partial net_{t-1}}=\left[\begin{array}{cccc} \frac{\partial s_{1}^{t-1}}{\partial net_{1}^{t-1}} & \frac{\partial s_{1}^{t-1}}{\partial net_{2}^{t-1}} & \cdots & \frac{\partial s_{1}^{t-1}}{\partial net_{n}^{t-1}}\\ \frac{\partial s_{2}^{t-1}}{\partial net_{1}^{t-1}} & \frac{\partial s_{2}^{t-1}}{\partial net_{2}^{t-1}} & \cdots & \frac{\partial s_{2}^{t-1}}{\partial net_{n}^{t-1}}\\ \vdots & \vdots & \vdots & \vdots \\ \frac{\partial s_{n}^{t-1}}{\partial net_{1}^{t-1}} & \frac{\partial s_{n}^{t-1}}{\partial net_{2}^{t-1}} & \cdots & \frac{\partial s_{n}^{t-1}}{\partial net_{n}^{t-1}} \end{array}\right]=\left[\begin{array}{cccc} f^{\prime }(net_{1}^{t-1}) & 0 & \cdots & 0\\ 0 & f^{\prime }(net_{2}^{t-1}) & 0 & 0\\ \vdots & \vdots & \ddots & \vdots \\ 0 & 0 & \cdots & f^{\prime }(net_{n}^{t-1}) \end{array}\right]=diag\left[f^{\prime }(net_{t-1})\right] \end{array}$$

(20) $$\begin{array}{l} \frac{\partial net_{t}}{\partial net_{t-1}}=\frac{\partial net_{t}}{\partial s_{t-1}}\frac{\partial s_{t-1}}{\partial net_{t-1}}\\ \begin{array}{ccc} & & = \end{array}Wdiag\left[f^{\prime }(net_{t-1})\right]\\ \begin{array}{ccc} & & = \end{array}\left[\begin{array}{cccc} w_{11}f^{\prime }(net_{1}^{t-1}) & w_{12}f^{\prime }(net_{2}^{t-1}) & \cdots & w_{1n}f^{\prime }(net_{n}^{t-1})\\ w_{21}f^{\prime }(net_{1}^{t-1}) & w_{22}f^{\prime }(net_{2}^{t-1}) & \cdots & w_{2n}f^{\prime }(net_{n}^{t-1})\\ \vdots & \vdots & \vdots & \vdots \\ w_{n1}f^{\prime }(net_{1}^{t-1}) & w_{n2}f^{\prime }(net_{1}^{t-1}) & \cdots & w_{nn}f^{\prime }(net_{1}^{t-1}) \end{array}\right] \end{array}$$

Therefore, $\delta_{k}^{T}$, which is the error per neuron, can be derived as:

(21) $$\begin{array}{l} \delta_{k}^{T}=\frac{\partial E}{\partial net_{k}}\\ \begin{array}{cc} & =\frac{\partial E}{\partial net_{t}} \end{array}\frac{\partial net_{t}}{\partial net_{k}}\\ \begin{array}{cc} & = \end{array}\frac{\partial E}{\partial net_{t}}\frac{\partial net_{t}}{\partial net_{t-1}}\frac{\partial net_{t-1}}{\partial net_{t-2}}\cdots \frac{\partial net_{k+1}}{\partial net_{k}}\\ \begin{array}{cc} & =Wdiag[f^{\prime } \end{array}(net_{t-1})]Wdiag\left[f^{\prime }(net_{t-2})\right]\cdots Wdiag\left[f^{\prime }(net_{k})\right]\delta_{t}^{l}\\ \begin{array}{cc} & = \end{array}\delta_{t}^{T}\prod_{i=k}^{t-1}Wdiag\left[f^{\prime }(net_{i})\right] \end{array}$$

in which E, k and l are the loss function, the initial moment and the ordinal number of the network layer, respectively. Just as with an ordinary multi-layer perceptron (MLP), the forward propagation of RNN between network layers can be written as:

(22) $$\begin{array}{l} net_{t}^{l}=Ua_{t}^{l-1}+Ws_{t-1}\\ a_{t}^{l-1}=f^{l-1}(net_{t}^{l-1}) \end{array}$$

Similarly, the relationship between two adjacent layers is:

(23) $$\begin{array}{l} \frac{\partial net_{t}^{l}}{\partial net_{t}^{l-1}}=\frac{\partial net^{l}}{\partial a_{t}^{l-1}}\frac{\partial a_{t}^{l-1}}{\partial net_{t}^{l-1}}\\ \begin{array}{ccc} & & = \end{array}Udiag\left[f^{\prime l-1}(net_{t}^{l-1})\right] \end{array}$$

The derivation process is the same as with that of Equation (19) to Equation (21). In this way, the gradient of each network layer can be detailed as:

(24) $$\begin{array}{l} {\left(\delta_{t}^{l-1}\right)}^{T}=\frac{\partial E}{\partial net_{t}^{l-1}}\\ \begin{array}{ccc} & & = \end{array}\frac{\partial E}{\partial net_{t}^{l}}\frac{\partial net_{t}^{l}}{\partial net_{t}^{l-1}}\\ \begin{array}{ccc} & & = \end{array}{\left(\delta_{t}^{l}\right)}^{T}Udiag\left[f^{\prime l-1}(net_{t}^{l-1})\right] \end{array}$$

As can be seen from the foregoing, $net_{t}$ is the key intermediate quantity in forward and back propagation. Furthermore, the expanded form of $net_{t}$ can be detailed as:

(25) $$\begin{array}{l} \left[\begin{array}{c} net_{1}^{t}\\ net_{2}^{t}\\ \vdots \\ net_{n}^{t} \end{array}\right]=Ux_{t}+Ws_{t-1}\\ \begin{array}{ccc} & & = \end{array}Ux_{t}+\left[\begin{array}{cccc} W_{11} & W_{12} & \cdots & W_{1n}\\ W_{21} & W_{22} & \cdots & W_{2n}\\ \vdots & \vdots & \vdots & \vdots \\ W_{n1} & W_{n2} & \cdots & W_{nn} \end{array}\right]\left[\begin{array}{c} s_{1}^{t-1}\\ s_{2}^{t-1}\\ \vdots \\ s_{n}^{t-1} \end{array}\right]\\ \begin{array}{ccc} & & = \end{array}Ux_{t}+\left[\begin{array}{cccc} W_{11}s_{1}^{t-1} & W_{12}s_{2}^{t-1} & \cdots & W_{1n}s_{n}^{t-1}\\ W_{21}s_{1}^{t-1} & W_{22}s_{2}^{t-1} & \cdots & W_{2n}s_{n}^{t-1}\\ \vdots & \vdots & \vdots & \vdots \\ W_{n1}s_{1}^{t-1} & W_{n2}s_{2}^{t-1} & \cdots & W_{nn}s_{n}^{t-1} \end{array}\right] \end{array}$$

The gradient of the loss function *E* to the weight *W* can be written as:

(26) $$\begin{array}{l} \frac{\partial E}{\partial w_{ji}}=\frac{\partial E}{\partial net_{j}^{t}}\frac{\partial net_{j}^{t}}{\partial w_{ji}}\\ \begin{array}{cc} & =\delta_{j}^{t} \end{array}s_{i}^{t-1} \end{array}$$

Hence, the gradient of *W* at time *t* is:

(27) $$\nabla_{Wt}E=\left[\begin{array}{cccc} \delta_{1}^{t}s_{1}^{t-1} & \delta_{1}^{t}s_{2}^{t-1} & \cdots & \delta_{1}^{t}s_{n}^{t-1}\\ \delta_{2}^{t}s_{1}^{t-1} & \delta_{2}^{t}s_{2}^{t-1} & \cdots & \delta_{2}^{t}s_{n}^{t-1}\\ \vdots & \vdots & \vdots & \vdots \\ \delta_{n}^{t}s_{1}^{t-1} & \delta_{n}^{t}s_{2}^{t-1} & \cdots & \delta_{n}^{t}s_{n}^{t-1} \end{array}\right]$$

Further, the sum of the gradients of *W* at each time instant can be given by:

(28) $$\begin{array}{l} \nabla_{W}E=\sum_{i=1}^{t}\nabla_{W_{i}}E\\ \begin{array}{cc} & = \end{array}\left[\begin{array}{cccc} \delta_{1}^{t}s_{1}^{t-1} & \delta_{1}^{t}s_{2}^{t-1} & \cdots & \delta_{1}^{t}s_{n}^{t-1}\\ \delta_{2}^{t}s_{1}^{t-1} & \delta_{2}^{t}s_{2}^{t-1} & \cdots & \delta_{2}^{t}s_{n}^{t-1}\\ \vdots & \vdots & \vdots & \vdots \\ \delta_{n}^{t}s_{1}^{t-1} & \delta_{n}^{t}s_{2}^{t-1} & \cdots & \delta_{n}^{t}s_{n}^{t-1} \end{array}\right]+\cdots +\left[\begin{array}{cccc} \delta_{1}^{1}s_{1}^{0} & \delta_{1}^{1}s_{2}^{0} & \cdots & \delta_{1}^{1}s_{n}^{0}\\ \delta_{2}^{1}s_{1}^{0} & \delta_{2}^{1}s_{2}^{0} & \cdots & \delta_{2}^{1}s_{n}^{0}\\ \vdots & \vdots & \vdots & \vdots \\ \delta_{n}^{1}s_{1}^{0} & \delta_{n}^{1}s_{2}^{0} & \cdots & \delta_{n}^{1}s_{n}^{0} \end{array}\right] \end{array}$$

Equally, the calculation of the weight *u* using the loss function can be described as:

(29) $$\begin{array}{l} \nabla_{U_{t}}E=\left[\begin{array}{cccc} \delta_{1}^{t}x_{1}^{t} & \delta_{1}^{t}x_{2}^{t} & \cdots & \delta_{1}^{t}x_{m}^{t}\\ \delta_{2}^{t}x_{1}^{t} & \delta_{2}^{t}x_{2}^{t} & \cdots & \delta_{2}^{t}x_{m}^{t}\\ \vdots & \vdots & \vdots & \vdots \\ \delta_{n}^{t}x_{1}^{t} & \delta_{n}^{t}x_{2}^{t} & \cdots & \delta_{n}^{t}x_{m}^{t} \end{array}\right]\\ \nabla_{U}E=\sum_{i=1}^{t}\nabla_{U_{t}}E \end{array}$$

**Figure 3:**
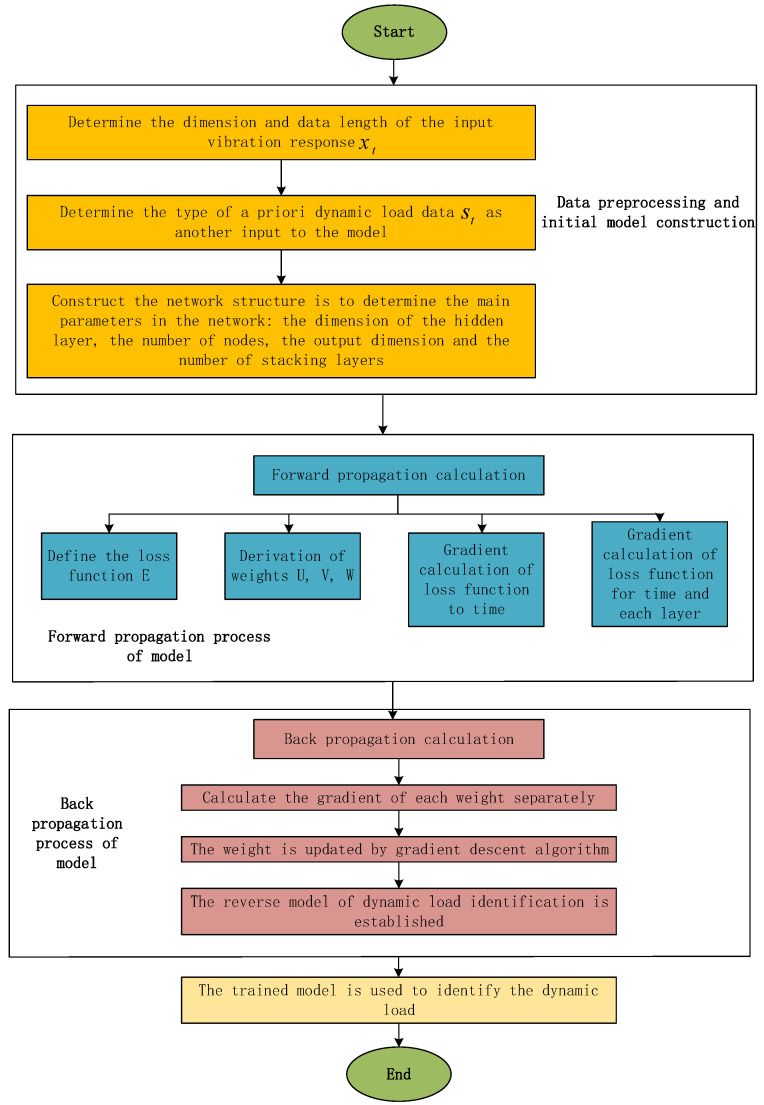
Dynamic load identification process based on RNN.

Finally, the weights are updated using a gradient descent algorithm. The fundamental process of our work is summarized in Figure 3.

Taking sinusoidal excitation as an example, using the amplitude at time t as the previous information from which to infer the amplitude at time t + 1 is the continuous process by which one can predict the dynamic load with the RNN model. However, vibration data often have a long time record where the excitation amplitude and the frequency will change with time. Under these circumstances, the simple RNN model is no longer suitable for the dynamic load identification process.

The method proposed here is based on LSTM, which is a variant of RNN. LSTM can save a long-term time record, which makes it possible to establish a relationship between the vibration response information and the dynamic load for the entire time domain. This feature enables the use of the change over time under a certain frequency and amplitude as a training data set to identify the dynamic loads under different frequencies and amplitudes. Moreover, LSTM can also better avoid the gradient disappearance problem caused by lengthy time histories compared to RNN.

### 2.3. Long Short-Term Memory Implementation

**Figure 4:**
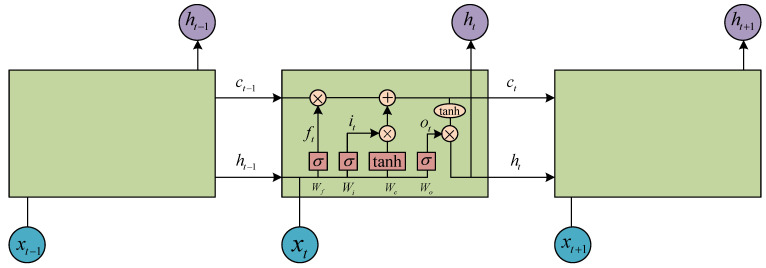
Structure of LSTM.

In vibration tests, the sampling rate is generally large and the acquisition time is long. Therefore, a vibration time series is often classified as a long series. In the process of calculating weight updates, the gradient will disappear because time-domain vibration data is usually a long time series [64]. Furthermore, the gradient will explode due to the increase in the number of neural network layers [65]. Consequently, the use of long short-term memory is necessary in our work. The LSTM layer is shown in Figure 4.

In contrast to RNN, LSTM neurons add a forgetting gate, a memory gate, an input gate and an output gate. In Figure 4, $f_{t},\;i_{t},\;c_{t},\;o_{t}$ and $h_{t}$ are outputs of the forgetting gate, the input gate, the combination of forgetting and input gates, the output gate and the final result, respectively. σ and tanh are the sigmoid function and hyperbolic tangent function, respectively. Furthermore, $W_{f},\;W_{i},\;W_{c}$ and $W_{o}$ are the weights of each part. These outputs can be written as:

(30) $$\begin{array}{l} f_{t}=\sigma (W_{f}\left[h_{t-1},x_{t}\right]+b_{f})\\ i_{t}=\sigma (W_{i}\left[h_{t-1},x_{t}\right]+b_{i})\\ c_{t}=f_{t}*c_{t-1}+i_{t}*\tanh(W_{c}\left[h_{t-1},x_{t}\right]+b_{c})\\ o_{t}=\sigma (W_{o}\left[h_{t-1},x_{t}\right]+b_{o})\\ h_{t}=o_{t}*\tanh(c_{t}) \end{array}$$

in which $b_{f},\;b_{i},\;b_{c}\;and\;b_{0}$ are the biases. Thus, the parameters to be learned for LSTM training are the weights and the biases in the above. Just as with the RNN derivation, we again use net as an intermediate variable. In this fashion, the intermediate output of each part can be obtained as:

(31) $$\begin{array}{l} net_{f,t}=W_{f}[h_{t-1},x_{t}]+b_{f}=W_{fh}h_{t-1}+W_{fx}x_{t}+b_{f}\\ net_{i,t}=W_{i}[h_{t-1},x_{t}]+b_{i}=W_{ih}h_{t-1}+W_{ix}x_{t}+b_{i}\\ net_{c,t}=W_{c}[h_{t-1},x_{t}]+b_{c}=W_{ch}h_{t-1}+W_{cx}x_{t}+b_{c}\\ net_{o,t}=W_{o}[h_{t-1},x_{t}]+b_{o}=W_{oh}h_{t-1}+W_{ox}x_{t}+b_{o} \end{array}$$

Moreover, the chain structure of LSTM is similar to that of RNN. Finally, the error transmitted from time *t* to any time *k* can be depicted as:

(32) $$\delta_{k}^{T}=\prod_{j=k}^{t-1}\delta_{o,j}^{T}W_{oh}+\delta_{f,j}^{T}W_{fh}+\delta_{i,j}^{T}W_{ih}+\delta_{\tilde{c},j}^{T}W_{ch}$$

where the error of each part can be detailed as:

(33) $$\begin{array}{l} \delta_{o,t}^{T}=\delta_{t}^{T}*\tanh(c_{t})*o_{t}*(1-o_{t})\\ \delta_{f,t}^{T}=\delta_{t}^{T}*o_{t}*(1-\tanh{\left(c_{t}\right)}^{2})*c_{t-1}*f_{t}*(1-f_{t})\\ \delta_{i,t}^{T}=\delta_{t}^{T}*o_{t}*(1-\tanh{\left(c_{t}\right)}^{2})*{\tilde{c}}_{t}*i_{t}*(1-i_{t})\\ \delta_{\tilde{c},t}^{T}=\delta_{t}^{T}*o_{t}*(1-\tanh{\left(c_{t}\right)}^{2})*i_{t}*(1-{\tilde{c}}^{2})\\ {\tilde{c}}_{t}=\tanh(W_{c}\left[h_{t-1},x_{t}\right]+b_{c}) \end{array}$$

Similarly, the gradient between layers can be written as:

(34) $$\frac{\partial E}{\partial net_{t}^{l-1}}=(\delta_{f,t}^{T}W_{fx}+\delta_{i,t}^{T}W_{ix}+\delta_{\tilde{c},t}^{T}W_{ix}+\delta_{o,t}^{T}W_{ox})*f^{\prime }(net_{t}^{l-1})$$

Eventually, the backpropagation through time (BPTT) algorithm is used to update the weight and the bias to complete the training of the model, and refers this to the RNN.

## 3. Numerical Studies

The dynamic load identification steps of a beam structure based on RNN are:

- Step A: Establish the deep network with the BLSTM layers, LSTM layers and full connection layers.
- Step B: Two groups of vibration response data are prepared. The first is the vibration response under an unknown dynamic load which is to be identified. The second is the vibration response under a known dynamic load which is different from the first group and used for training. The proposed algorithm is then trained using the second group with the known dynamic load, while the responses obtained from the first group are used to identify the unknown load. Furthermore, these data groups are divided into a training set, a verification set and a test set on the basis of equipment computational ability.
- Step C: The backpropagation through time (BPTT) algorithm is used as a model training method to update the parameters of the model. In addition, the initial learning rate and batch size are set in the light of available computer memory. To accelerate the training speed, the training process is run on a GPU device.
- Step D: The new vibration response data are used to test the identification effect of the model. In this paper, two methods are introduced to appraise the effect of identification: the peak relative error method (PREM) and the signal-to-noise ratio (SNR). PREM is the maximum value of the peak error of the load identification result and can be written in Equation (35) as:

(35) $$PREM(X,Y)=\frac{\left|\max Y(i)-\max X(i)\right|}{\max X(i)}\times 100\%$$

in which X(i) and Y(i) are the actual load and the identified load signal, respectively. Moreover, SNR is the signal-to-noise ratio of dynamic load identification results, which describes the overall effect of dynamic load identification. The calculation of SNR can be detailed as:

(36) $$SNR(X,Y)=10\log_{10}\left[\frac{\sum_{i=1}^{ns}X{\left(i\right)}^{2}}{\sum_{i=1}^{ns}{\left(X\left(i\right)-Y\left(i\right)\right)}^{2}}\right]$$

where ns is the number of acquisition points in the analysis period.

Additionally, we compare the results based on RNN with MLP. The two methods use the same vibration response data to identify the same dynamic load. Furthermore, the best MLP structure is selected to identify the dynamic load and the overlapping parameters are set to be the same as MLP.

### 3.1. Model Parameters

We analyze the dynamic load identification cases of the simply supported beam under three kinds of excitation: sinusoidal dynamic load, impact dynamic load or random load. All the loads are applied at one point, as shown in Figure 1. The simply supported beam is 5 m long, 0.25 m wide and 0.05 m thick. Moreover, the elastic modulus, Poisson’s ratio and the density of the simply supported beam are 210 Gpa, 0.31 and 7800 $\mathrm{Kg}/m^{3}$, respectively, as shown in Table 1. In addition, the simply supported beam is divided into 10 sections and 11 nodes. Table 2 describes the distance from each node to the coordinate’s origin. We have carried out modal analysis on the simply supported beam and the first ten natural frequencies are shown in Table 3.

### 3.2. Considered Cases

In order to evaluate the proposed method, three numerical cases are analyzed using RNN and MLP. Furthermore, three SNRs (10 dB, 20 dB, 30 dB) are added to the input vibration response data to compare the robustness of RNN and MLP. The dynamic load parameters of the three cases are:

Case 1: A sinusoidal excitation F=85sin(30πt) is applied at *a* = 1.5 m. The time interval is 0.0001 s and the full considered time span is 1 s. The sinusoidal load function used for training is F=50sin(15πt).

Case 2: An impact excitation is applied at *a =* 1.5 m and the function is:

$$F=\{\begin{array}{c} 30\sin\left(50\pi t\right),t\epsilon \left[0.56,0.58\right]\\ 50\sin\left(50\pi t\right),t\epsilon \left[0.64,0.66\right] \end{array}.$$

The time interval is 0.0001 s and the full considered time span is 0.22 s. The dynamic load data for training are made up of continuous hammering from 0 s to 0.56 s using F and the vibration response data for training are obtained under this load.

Case 3: A random excitation is applied at *a =* 1.5 m. The variance of the excitation is 100 and the mean value is 0. Moreover, the time interval is 0.0001 s and the full considered time span is 0.2 s. The dynamic load used for training is a random excitation with a variance of 25 and a mean value of 0 while the vibration response data for training are obtained under this load. The parameters of the three dynamic loads are described in detail in Table 4.

The RNN used for these cases has one BLSTM layer, one LSTM layer and two fully connected layers. The BLSTM is a form of LSTM that allows the current output to be obtained by the combination of the previous output and the future output. Consequently, we added the BLSTM layer based on the statistical regularity of conventional excitation data. In order to reflect the comparison, the number of neurons and layers in the fully connected layers in RNN is the same as that in MLP. Dropout regularization is used to improve the generalization ability of the model. The computations are performed on an intel i5-9300H CPU and an NVIDIA GTX1650 GPU. In addition, we use both the GPU and the CPU to calculate the dynamic loads. The GPU is only used to improve the computing efficiency compared with the CPU, and its influence on the calculation accuracy is insignificant and can be ignored. Hence, the identification results are only presented with the GPU in this paper.

### 3.3. Identification Results and Comparisons

**Figure 5:**
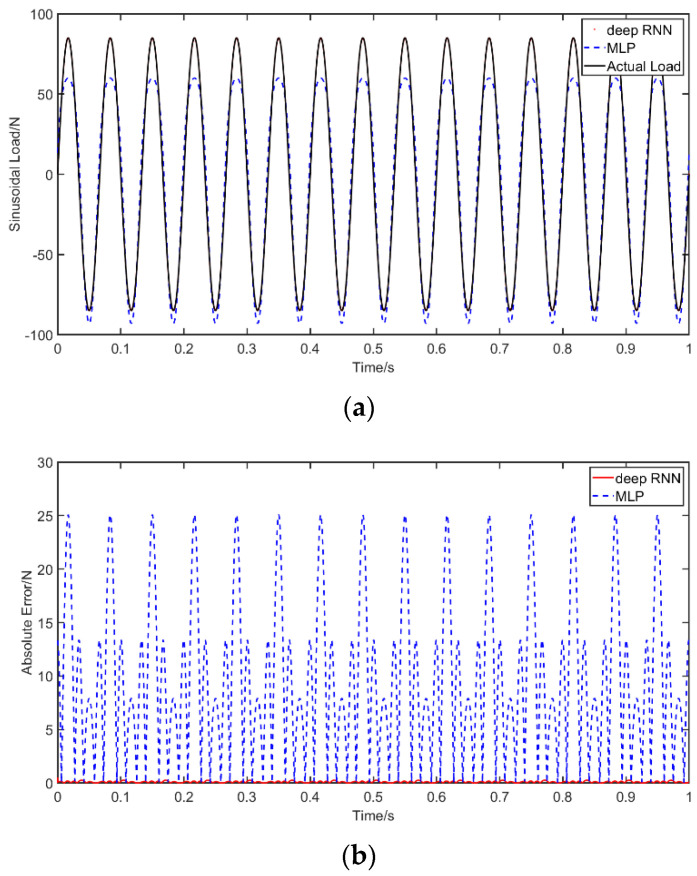
Identification results from sinusoidal excitation: (**a**) comparison of results of the use of deep RNN and MLP with a sinusoidal excitation; (**b**) comparison of errors of the use of deep RNN and MLP with a sinusoidal excitation.

Case 1: The data concerning the sinusoidal excitation applied on the beam was recovered with the RNN and MLP networks that were trained using the method previously described above. The results without noise and the absolute errors of identification result are shown in Figure 5, in which we show comparisons of deep RNN, MLP and the actual load, as well as the absolute errors of deep RNN and MLP. The abscissa unit is s and the ordinate unit is N.

**Figure 6:**
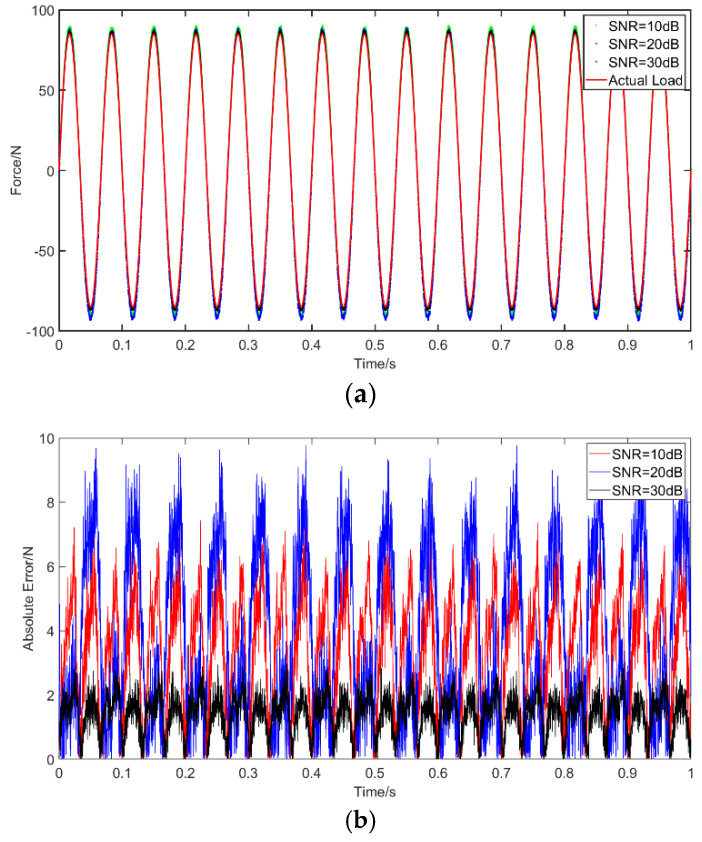
Identification results from sinusoidal excitation with noises: (**a**) comparison of results of the use of deep RNN with a sinusoidal excitation under three noises; (**b**) comparison of errors of the use of deep RNN under noises compared with an actual load.

Figure 6 presents the results with different noise levels, specifically, 10 dB, 20 dB and 30 dB, and shows comparisons for deep RNN under these three noises, and the errors compared with the actual load. The abscissa unit is s and the ordinate unit is N. Table 5 describes the PREM and SNR of RNN and MLP without noise as well as PREM and SNR of RNN for the different considered noise levels. The results show that the error of the sinusoidal load identification based on RNN is smaller than that based on MLP without noise. Moreover, PREM and SNR are within the acceptable range. After adding the noise, the error increases significantly when compared to a noise-free environment. Under 10 dB of noise, the PREM reaches a maximum of 6.18% and the SNR is 24.50. Overall, however, even when the noise is considered, the errors remain within an acceptable level for engineering accuracy. The obtained results indicate the accuracy and the robustness of the proposed method.

**Figure 7:**
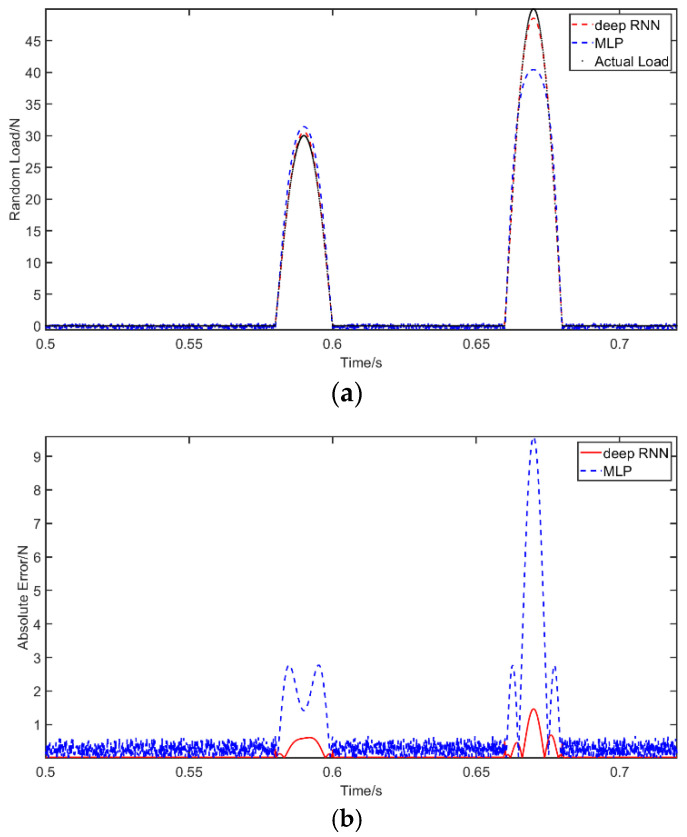
The identification results from impact excitation: (**a**) comparison of results of the use of deep RNN and MLP with an impact excitation; (**b**) comparison of errors of the use of deep RNN and MLP with an impact excitation.

**Figure 8:**
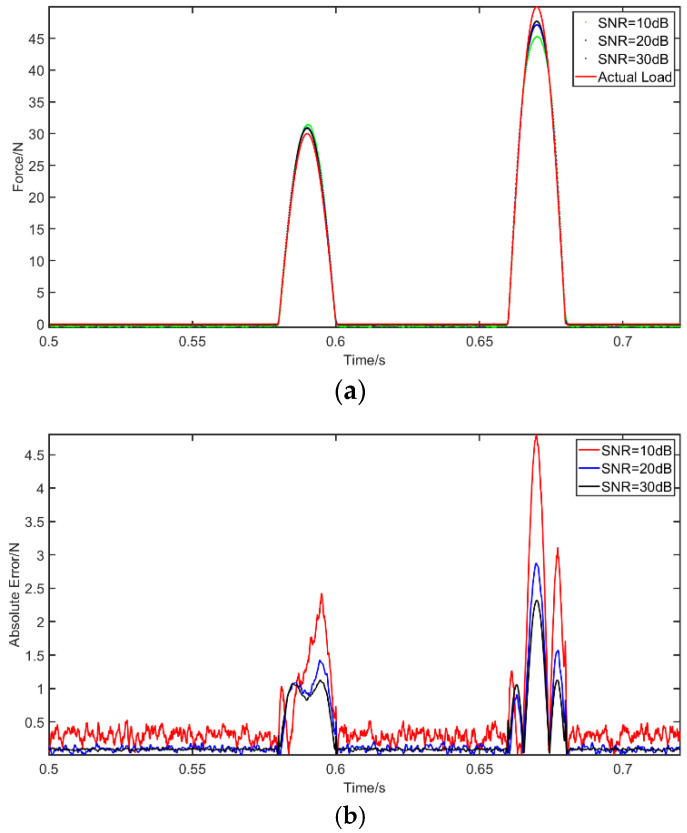
The identification results from impact excitation with noises: (**a**) comparison of results of the use of deep RNN with an impact excitation under three noises; (**b**) comparison of errors of the use of deep RNN under noises compared with actual load.

Case 2: A half sine wave $F=\{\begin{array}{c} 30\sin\left(50\pi t\right),t\epsilon \left[0.56,0.58\right]\\ 50\sin\left(50\pi t\right),t\epsilon \left[0.64,0.66\right] \end{array}$ is used as an excitation on the beam at *a =* 1.5 m. Again, we first compare the results of RNN and MLP where no noise is used. Next, the resilience of the RNN method is evaluated under noisy conditions. The presentation of the results is similar to Case 1. Figure 7 shows the comparison of the RNN and MLP identifications. The performance of RNN under noisy conditions are presented in Figure 8. Moreover, Table 6 shows the PREM and SNR for the different considered solutions. It can be inferred that the identification results of MLP concerning the impact load are unsatisfactory. This is especially so compared to the high accuracy of RNN and its ability to recover the load curve with good precision. Clearly, the MLP results capture the time instants of the two impacts, but the amplitude of the largest impact is significantly missed. Additionally, the robustness of the RNN model to the impact load is acceptable, given that its PREM reaches the maximum value of 9.37% at 10 dB, while the SNR is 22.52.

**Figure 9:**
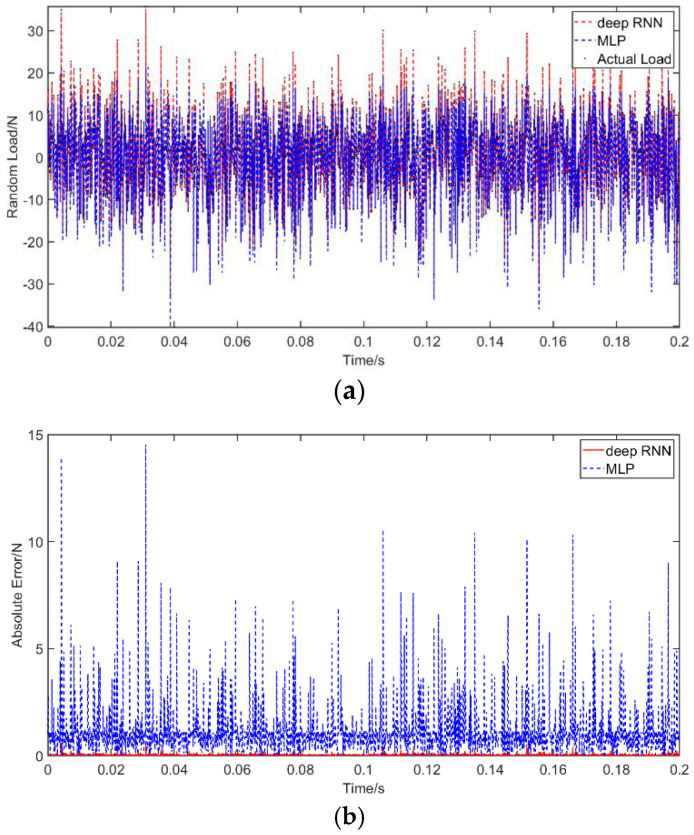
Identification results from random excitation: (**a**) comparison of results of the use of deep RNN and MLP with a random excitation; (**b**) comparison of errors of the use of deep RNN and MLP with a random excitation.

**Figure 10:**
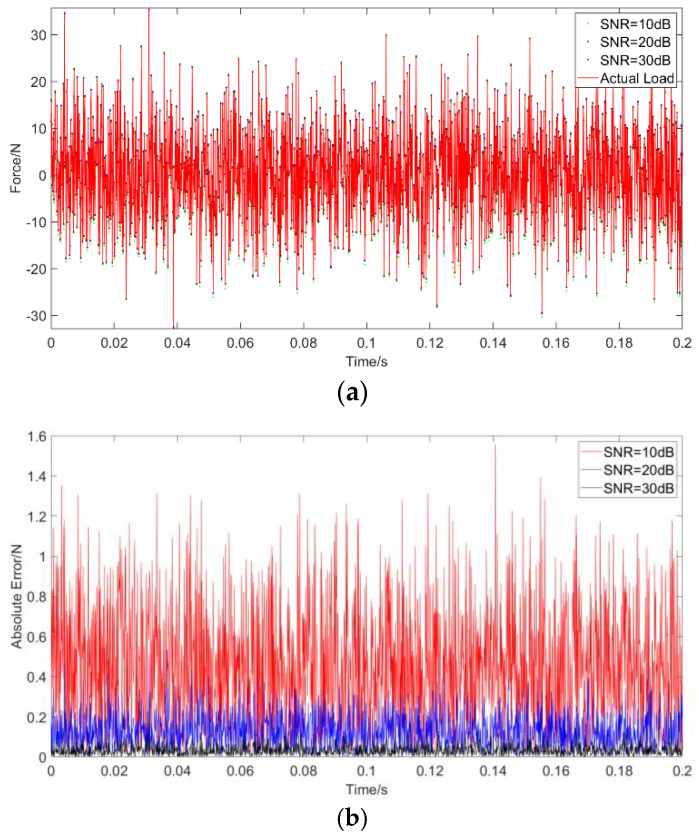
Identification results from random excitation with noises: (**a**) comparison of results of the use of deep RNN with a random excitation under three noises; (**b**) comparison of errors of the use of deep RNN under noises compared with an actual load.

Case 3: The considered random load applied in this example is a Gaussian white noise with a variance of 100 and a mean value of 0. The dynamic load is again applied at *a =* 1.5 m. As with before, the results of RNN and MLP are compared for a noise-free environment, while only the performance of RNN is evaluated under noise. The results without noise are plotted in Figure 9 and the results with noise in Figure 10, in which the abscissa unit is s and the ordinate unit is N. The PREM and SNR for different solutions are presented in Table 7. The results are consistent with before and indicate the advantages of RNN in the time domain compared with MLP. The RNN shows a high level of identification accuracy in noise-free environments. When adding noise to the load identification input, the RNN results remain acceptable. The PREM reaches the maximum value of 1.69% at 10 dB, while the SNR is 25.29 under a 10 dB noise level.

## 4. Experimental Results

### 4.1. Experimental Setting

**Figure 11:**
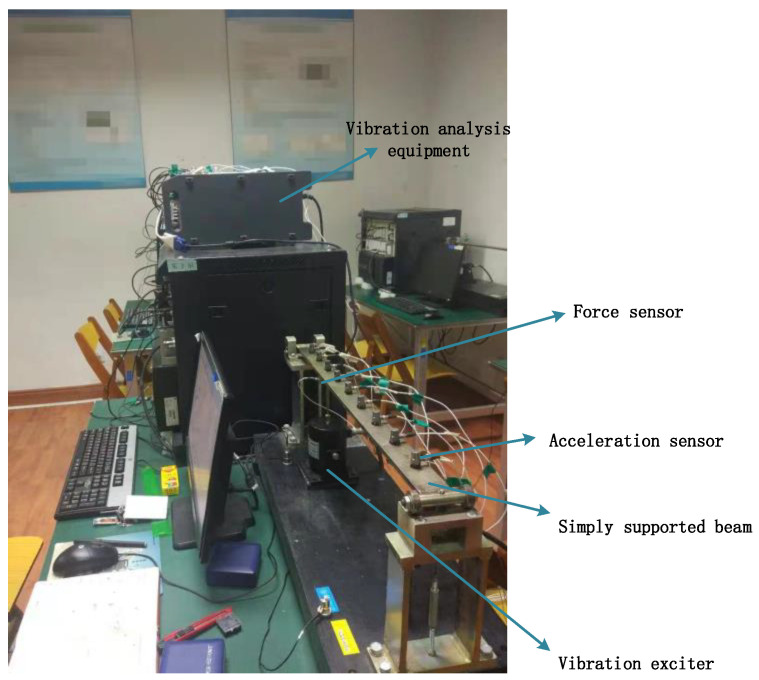
Experimental settings.

In this section, experimental results are used to further validate the reliability and feasibility of the proposed approach. A simply supported beam is set up with vibration analysis equipment, acceleration and force sensors, as well as vibration exciters. The test setting is shown in Figure 11. Nine acceleration sensors (PCB Unidirectional acceleration sensor) are arranged on the beam to collect the vibration response information. A vibration exciter (NTS Vibration exciter) is applied 0.21 m away from the left end of the beam and a vibration analyzer (M+P VibMoblie) is used to collect vibration data, as can be seen in the figure. Two types of load are applied, namely, sinusoidal excitation and random excitation. Additionally, the vibration responses under these two types of excitations are measured at the same location where the exciter is applied, i.e., 0.21 m from the left end. The function of the sinusoidal excitation is F=1.8sin(150πt) and the random excitation is again Gaussian white noise with a variance of 100 and a mean value of 0. It should be noted that, for the training purposes, the sinusoidal excitation is F=10sin(100πt) and the random excitation is Gaussian white noise with 25 variance and a mean value of 0. The sample rate is set to 6400. The time span of the calculations for the sinusoidal excitation and the random excitation is fixed at 0.5 s. Moreover, the experimental validation measures, PREM and SNR, are used to evaluate the identification accuracy. The parameters of the simply supported beam are shown in Table 8 and the parameters of the dynamic loads in Table 9.

### 4.2. Experimental Results

**Figure 12:**
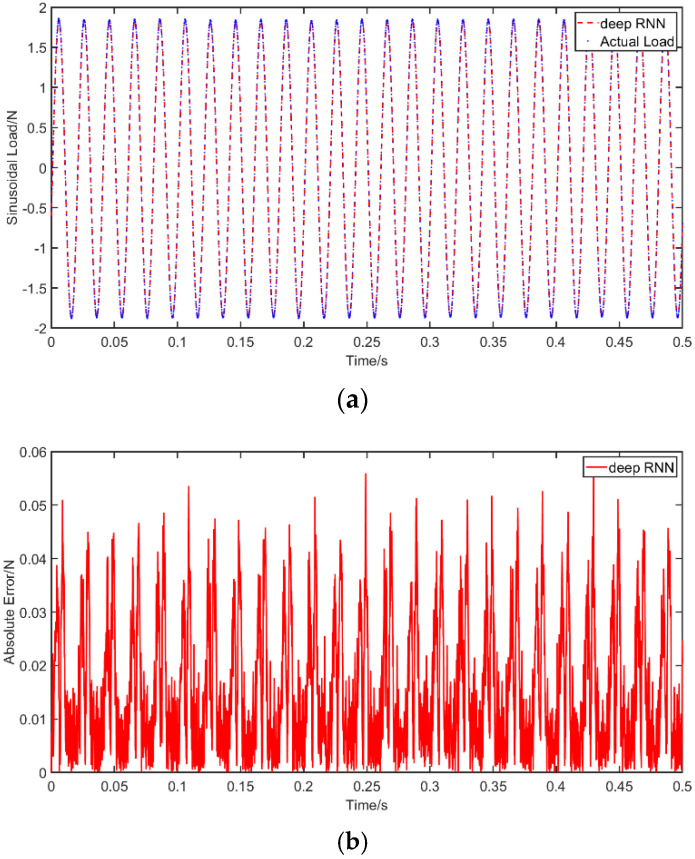
Identification results for sinusoidal excitation from the practical experiment: (**a**) results of the use of deep RNN compared with actual load with a sinusoidal excitation in an experiment; (**b**) absolute error of the use of deep RNN with a sinusoidal excitation in an experiment.

**Figure 13:**
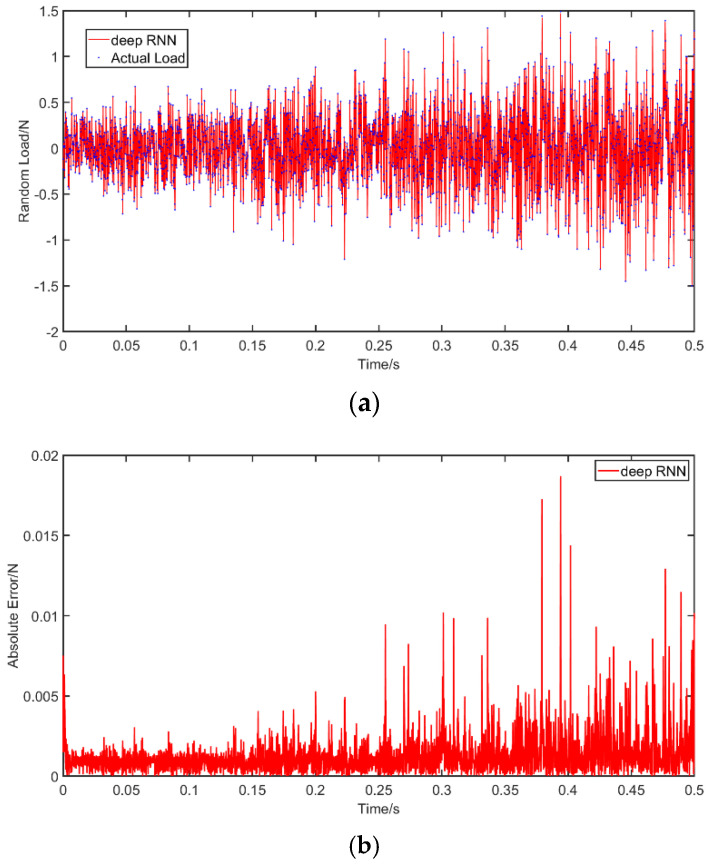
Identification results for random excitation from the practical experiment: (**a**) results of the use of deep RNN compared with actual load with a random excitation in an experiment; (**b**) absolute error of the use of deep RNN with a random excitation in an experiment.

The excitations mentioned in Table 9 were applied to the simply supported beam. The vibration responses that were obtained were transferred through the proposed model as described in Section 3.2. The results of the sinusoidal excitation are presented in Figure 12 and those of the random excitation in Figure 13, in which the abscissa unit is s and the ordinate unit is N, similar to the presentation of the results of the numerical analysis. The PREM and SNR for the different obtained solutions are displayed in Table 10. The results again reflect the high reliability and accuracy of the proposed model. The PREMs of the sinusoidal excitation and random excitation are 1.27% and 1.26%, respectively, while the SNRs of these two excitations are 36.42 and 46.28. The precision and robustness of the proposed method mean that it is of great practical value for many engineering applications. However, the proposed method requires a relatively large amount of vibration response data, which also means that an extended amount of time is needed to train the model. In addition, this method requires a priori data of the dynamic load, that is, historical data or similar data of the target’s dynamic load. For completely unknown dynamic loads, this method might face difficulties predicting the load accurately.

## 5. Implementation Factors

Taking the simply supported beam in Figure 11 with the sinusoidal excitations as an example, we now aim to perform a detailed analysis of the proposed method. We first evaluate the influence of the hyperparameters on the dynamic load identification results. The impact of the RNN models with different structures on the identification results is also studied. Then, we evaluate the proposed method of identifying dynamic loads under multi-points excitations. Finally, we check if the identification results are affected by the layouts or the measuring points. To this end, we build three models using different layers and adjust the hyperparameters, which consist of each neuron’s number and learning rate and the training time, and compare the performance relative to PREM and SNR of the obtained results.

### 5.1. Effect of Different Architectures and Hyperparameters

To discuss the impression of the model structure on the identification results, we constructed three different models: an RNN model (two layers without LSTM), an LSTM model (two layers with LSTM) and a BLSTM model (one BLSTM and one LSTM layer). Furthermore, the number of neurons, which affects the learning ability of the network, and the learning rate, which represents the calculation step size of the update algorithm, are changed to evaluate the influence of the hyperparameters on the identification results. All the considered variations and their relevant accuracy results are presented in Table 11. For reference we also show in the table the respective GPU and CPU times in minutes needed to perform the computations.

In general, the proposed approach remains effective for the different cases shown in the table. However, the changes in the structure and the hyperparameters shows a meaningful impact on the identification results, especially for the structure without LSTM. The value of PREM increases and of SNR decreases without LSTM. This behavior is consistent with the fact that RNN cannot deal with vanishing and exploding gradients. The model with BLSTM shows an improved identification ability but it also requires longer training times. It is to be noted that the BLSTM model is the structure considered in Section 3 and Section 4. Finally, the increase in the number of neurons and the reduction of the learning rate improve the identification accuracy. Nevertheless, using an excessive number of neurons will result in data being over-fitted, while a very low learning rate will prevent the convergence of the network’s gradients.

### 5.2. Effect of Multi-Point Excitations

**Figure 14:**
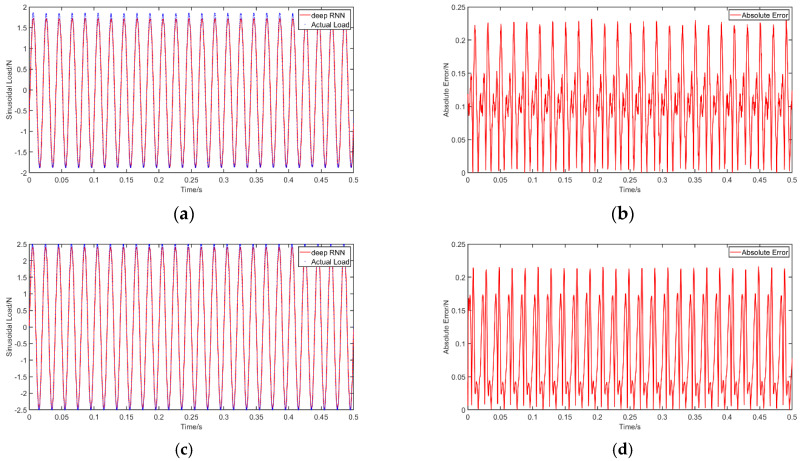
Identification results for multi-point excitation: (**a**) identification curve for Excitation 1; (**b**) absolute error for Excitation 1; (**c**) identification curve for Excitation 2; (**d**) absolute error for Excitation 2.

To evaluate the effect of multi-point excitations, we apply two sinusoidal loads on the simply supported beam considered Section 4. The first sinusoidal load (Excitation 1) is $F_{1}=1.8\sin\left(150\pi t\right)$ and applied at *a =* 0.21 m from the left support, while the second (Excitation 2) is $F_{2}=2.5\sin\left(100\pi t\right)$ and at *a =* 0.56 m. The BLSTM model in Section 5.1 is then used to identify the loads that are applied simultaneously. The identification results are presented in Figure 14 and the accuracy is shown in Table 12. Clearly, the identification results of the two-point excitation is worse than that under a single-point excitation, which is consistent with the research results for multi-point excitations in [66,67]. However, the proposed method can accurately identify the excitation curves, as can be seen in the figure. Moreover, the absolute error, PREM and SNR are acceptable.

### 5.3. Effect of Different Measuring Points

**Figure 15:**
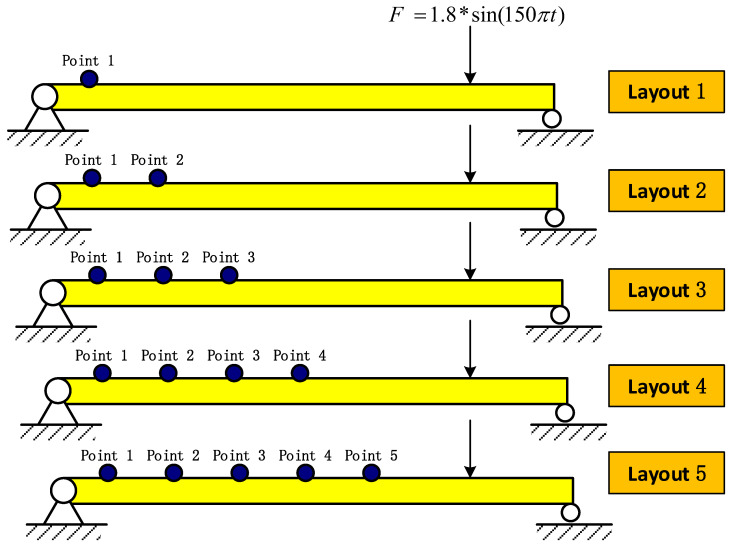
Different considered layouts for measurement points.

**Figure 16:**
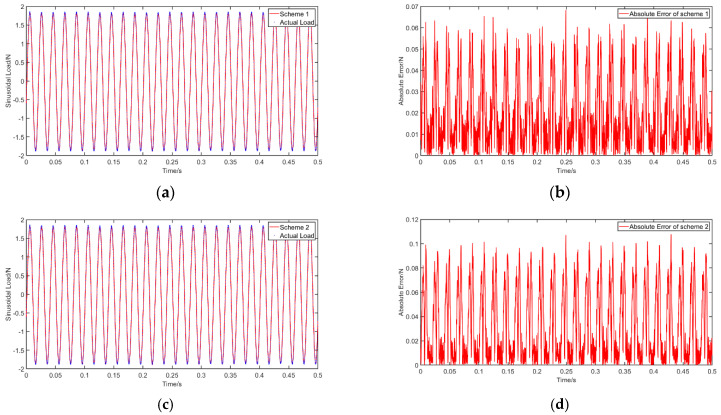

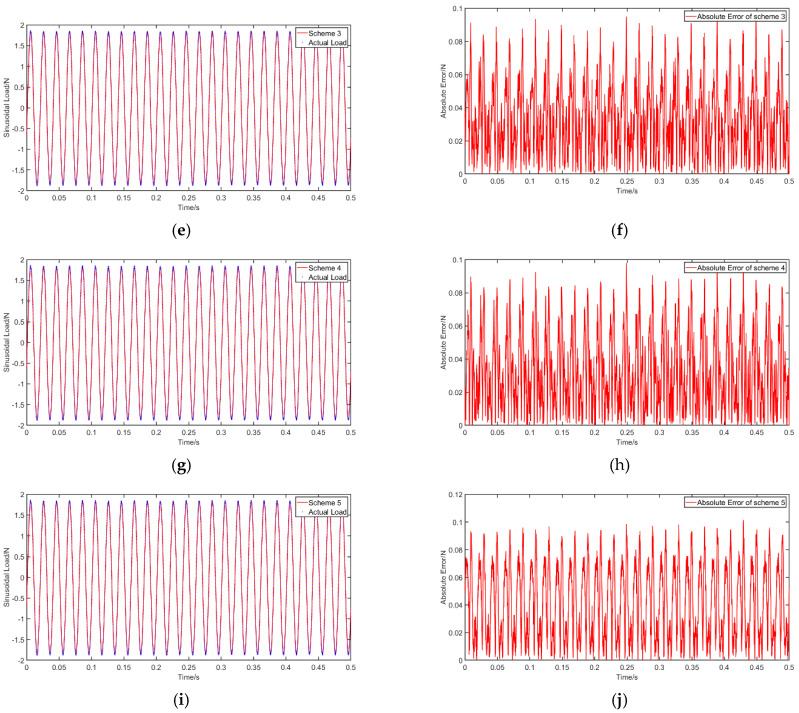
The identification results for layout of measuring points: (**a**) identification curve for layout 1; (**b**) absolute error for layout 1; (**c**) identification curve for layout 2; (**d**) absolute error for layout 2; (**e**) identification curve for layout 3; (**f**) absolute error for layout 3; (**g**) identification curve for layout 4; (**h**) absolute error for layout 4; (**i**) identification curve for layout 5; (**j**) absolute error for layout 5.

Finally, we want to evaluate the impact of using different numbers and positions of points to take the measurements on the accuracy. Thus, we assess the impact of choosing a specific measurement profile compared to others on the identification accuracy of RNN. We propose five layouts of measurement points on the simply supported beam defined in Section 4. Each layout is different based on the positions and the number of considered points, as shown in Figure 15. The distance from the measuring point to the left end, the distance from the excitation point to the right end and the distance between the measuring points are all fixed at 0.21 m. The BLSTM network defined in Section 5.1 is again used here. The impact of different layouts on the identification results is shown in Figure 16 and Table 13.

It can be inferred from the results that the identification accuracy is not affected by changing the measurement layout. Furthermore, the dynamic loads can be accurately identified even when using only one measuring point. In many engineering applications, this can be an important feature for the proposed approach, given that the accuracy of the RNN model is insensitive to the measurement layout. This feature can significantly reduce the complexity of the vibration measurements.

## 6. Conclusions

In this paper, a novel method based on a recurrent neural network is proposed for the dynamic load identification of a simply supported beam. The model is based on RNN and LSTM. The data needed to train and validate the model are created from different types of dynamic loads, i.e., sinusoidal, impact and random loads. The model is then used to identify the dynamic load using the vibration response from different excitations. The results show that the proposed method has a good identification accuracy and is reliable even when used with noisy measurements or when considering multiple excitations simultaneously. To evaluate the stability of the proposed algorithm, we also considered its performance using different network structures and different values for the hyperparameters. We finally analyze the sensitivity of the proposed algorithm to the number and the layout of the points where measurements are taken. Based on the presented results, the proposed model has the following advantages:

1. Compared with conventional methods, the proposed algorithm can avoid the need to solve the model parameters of the structure. This can significantly reduce the difficulty of dynamic load identification as assessing the dynamic properties of a structure cannot always be possible.
2. The presented results shows that the proposed algorithm for dynamic load identification is accurate, stable and robust.
3. The proposed method is suitable for single-point or multi-point excitations. Similarly, the method does not display sensitivity to changing the vibration measurement layouts.
4. Using different structures for the model network and the choice of the hyperparameters has a limited impact on the identification results. The choice of the structure and the hyperparameters can then be made based on balancing the required accuracy against the time available to train the network.

Despite the different advantages of models built with RNN for change-over-time applications, this work is the first to utilize such models to identify different dynamic loads applied to a simply supported beam. We hope the work can bring some fresh ideas into the dynamic load identification academic and industrial communities.

## Figures and Tables

**Table 1:** Parameters of the simply supported beam.

Parameters	Value
Length *l*	5 m
Width *a*	0.25 m
Thickness b	0.05 m
Elastic modulus E	210 GPa
Poisson’s ratio *ε*	0.31
Density *ρ*	$7800\;\mathrm{Kg}/m^{3}$

**Table 2:** Specific location of measuring points.

Measuring Point	1	2	3	4	5	6	7	8	9
Position (m)	0.5	1.0	1.5	2.0	2.5	3.0	3.5	4.0	4.5

**Table 3:** Natural frequency of the simply supported beam.

**Modal Order**	**1**	**2**	**3**	**4**	**5**
Frequency (Hz)	4.7	18.8	42.3	52.6	74.9
**Modal Order**	**6**	**7**	**8**	**9**	**10**
Frequency (Hz)	116.4	117.1	142.6	165.4	219.1

**Table 4:** Load conditions of the three considered cases.

Case	Dynamic Load	Excitation Parameters	Sampling Parameters
1	Sinusoidal	$x_{}=1.5m,F_{}=85*\sin(30\pi t)$ The excitation for training is $x=1.5m,F_{}=50*\sin(15\pi t)$	$\begin{array}{l} \Delta t=0.0001s,\\ t=1s \end{array}$
2	Impact	$x_{}=1.5m,F_{}=\{\begin{array}{c} 30*\sin(50\pi t),t\in [0.56,0.58]\\ 50*\sin(50\pi t),t\in [0.64,0.66] \end{array}$ The excitation for training is $x_{}=1.5m,F_{}=\{\begin{array}{c} \begin{array}{l} 30*\sin(50\pi t)\\ t\in [0.08,0.10],[0.24,0.26],[0.40,0.42] \end{array}\\ \begin{array}{l} 50*\sin(50\pi t)\\ t\in [0.16,0.18],[0.32,0.34],[0.48,0.50] \end{array} \end{array}$	$\begin{array}{l} \Delta t=0.0001s,\\ t=0.22s \end{array}$
3	Random	white Gaussian noise $x_{}=1.5m,\mathrm{var}iance=100,mean=0$ The excitation for training is white Gaussian noise $x_{}=1.5m,\mathrm{var}iance=25,mean=0$	$\begin{array}{l} \Delta t=0.0001s,\\ t=0.2s \end{array}$

**Table 5:** The evaluation of sinusoidal excitation identification results.

	No Noise		RNN with Noise		
	RNN	MLP	10 dB	20 dB	30 dB
PREM	0.22%	29.51%	6.18%	4.24%	2.66%
SNR	55.07	13.39	24.50	22.73	32.11

**Table 6:** Evaluation of impact excitation identification results.

	No Noise		RNN with Noise		
	RNN	MLP	10 dB	20 dB	30 dB
PREM	2.94%	19.16%	9.37%	5.70%	4.64%
SNR	34.10	17.15	22.52	27.01	28.62

**Table 7:** Evaluation of random excitation identification results.

	No Noise		RNN with Noise		
	RNN	MLP	10 dB	20 dB	30 dB
PREM	1.71%	40.65%	1.69%	1.43%	0.82%
SNR	43.27	14.85	25.29	35.98	47.20

**Table 8:** Parameters for the simply supported beam experiment.

Parameters	Value
Length *l*	0.7 m
Width *a*	0.04 m
Thickness b	0.008 m
Elastic modulus E	210 GPa
Poisson’s ratio *ε*	0.3
Density *ρ*	7800 kg/m^3^

**Table 9:** Load conditions for two cases of experiments.

Case	Dynamic Load	Excitation Parameters	Sampling Parameters
1	Sinusoidal	$x_{}=0.21m,F=1.8*\sin150(\pi t)$ The excitation for training is $x_{}=0.21m,F=10*\sin(100\pi t)$	$\Delta t=\frac{1}{6400}s,t=0.5s$
2	Random	white Gaussian noise $x_{}=0.21m,\mathrm{var}iance=100,mean=0$ The excitation for training is white Gaussian noise $x_{}=0.21m,\mathrm{var}iance=25,mean=0$	$\Delta t=\frac{1}{6400}s,t=0.5s$

**Table 10:** Evaluation of experimental results.

	Excitation	
	Sinusoidal Excitation	Random Excitation
PREM	1.27%	1.26%
SNR	36.42	46.28

**Table 11:** Identification results with changing structures and hyperparameters.

	Hyperparameter		Training Time by GPU(CPU) in min	PREM	SNR
	Number of Neurons	Learning Rate			
1BLSTM+ 1LSTM+ 2FC	128	0.01	18(25)	1.35%	33.28
		0.005	41(68)	1.27%	44.29
		0.001	52(86)	1.27%	45.42
	256	0.01	29(48)	1.25%	46.42
		0.005	58(77)	1.27%	45.20
		0.001	85(132)	1.22%	45.58
2LSTM+ 2FC	128	0.01	12(17)	2.86%	28.17
		0.005	28(36)	1.52%	35.74
		0.001	46(66)	1.48%	37.55
	256	0.01	25(40)	4.79%	30.74
		0.005	65(88)	2.87%	38.26
		0.001	85(125)	2.76%	38.65
2RNN+ 2FC	128	0.01	12(15)	8.78%	22.93
		0.005	21(32)	6.42%	25.66
		0.001	32(47)	7.29%	27.31
	256	0.01	22(25)	5.31%	23.75
		0.005	34(47)	4.10%	28.12
		0.001	41(56)	4.08%	31.07

**Table 12:** Evaluation of the results of multi-point excitation.

	Excitation	
	Excitation 1	Excitation 2
PREM	7.07%	3.52%
SNR	20.52	24.27

**Table 13:** Evaluation of the results of multi-point excitation.

	Different Layouts of Measuring Points				
	Layout 1	Layout 2	Layout 3	Layout 4	Layout 5
PREM	1.60%	2.42%	0.90%	2.35%	1.61%
SNR	34.02	29.41	30.47	30.32	28.43

## Data Availability

Data is available by requests and should be directed to the second co-author Jinhui Jiang (Jiangjinhui@nuaa.edu.cn).
